# An investigation of how relative precision of target encoding influences metacognitive performance

**DOI:** 10.3758/s13414-020-02190-0

**Published:** 2020-11-26

**Authors:** Sanne Kellij, Johannes Fahrenfort, Hakwan Lau, Megan A. K. Peters, Brian Odegaard

**Affiliations:** 1 Sociology Department, Rijksuniversiteit Groningen, Groningen, the Netherlands; 2 Developmental and Educational Unit, Institute of Psychology, Leiden University, Leiden, the Netherlands; 3 Department of Experimental and Applied Psychology, Institute Brain and Behavior Amsterdam (iBBA), VU University Amsterdam, Amsterdam, the Netherlands; 4 Department of Psychology, University of Amsterdam, Amsterdam, the Netherlands; 5 Amsterdam Brain and Cognition (ABC), University of Amsterdam, Amsterdam, the Netherlands; 6 Department of Psychology, University of California Los Angeles, Los Angeles, CA 90095-1563 USA; 7 Brain Research Institute, University of California Los Angeles, Los Angeles, CA 90095-1563 USA; 8 Department of Psychology, University of Hong Kong, Pokfulam Road, Pok Fu Lam, Hong Kong; 9 State Key Laboratory of Brain and Cognitive Sciences, University of Hong Kong, Pokfulam Road, Pok Fu Lam, Hong Kong; 10 Department of Bioengineering, University of California Riverside, Riverside, CA 92521 USA; 11 Department of Cognitive Sciences, University of California Irvine, Irvine, CA 92697 USA; 12 Department of Psychology, University of Florida, FL 32603 Gainesville, USA

**Keywords:** Metacognition, Signal Detection Theory, Attentional blink, Phase scrambling

## Abstract

Detection failures in perceptual tasks can result from different causes: sometimes we may fail to see something because perceptual information is noisy or degraded, and sometimes we may fail to see something due to the limited capacity of attention. Previous work indicates that metacognitive capacities for detection failures may differ depending on the specific stimulus visibility manipulation employed. In this investigation, we measured metacognition while matching performance in two visibility manipulations: phase-scrambling and the attentional blink. As in previous work, metacognitive asymmetries emerged: despite matched type 1 performance, metacognitive ability (measured by area under the ROC curve) for reporting stimulus absence was higher in the attentional blink condition, which was mainly driven by metacognitive ability in correct rejection trials. We performed Signal Detection Theoretic (SDT) modeling of the results, showing that differences in metacognition under equal type I performance can be explained when the variance of the signal and noise distributions are unequal. Specifically, the present study suggests that phase scrambling signal trials have a wider distribution (more variability) than attentional blink signal trials, leading to a larger area under the ROC curve for attentional blink trials where subjects reported stimulus absence. These results provide a theoretical basis for the origin of metacognitive differences on trials where subjects report stimulus absence, and may also explain previous findings where the absence of evidence during detection tasks results in lower metacognitive performance when compared to categorization.

## Introduction

Our daily lives require that we be able to detect the presence or absence of objects in the environment. For example, when driving a car, we may primarily be concerned with detecting objects that might interfere with the path of our vehicle. Potentially, there may be different reasons why we fail to detect something: sometimes we may not detect an object because our perception is *degraded* (due to fog, low light, or other factors), and other times we may not detect an object due to lapses in our *attention*. These two scenarios involving detection failures raise interesting questions: is our subjective sense of visual confidence in what we see in these scenarios roughly equivalent, or does it differ? And if it does differ, why might this be the case?

Kanai, Walsh, and Tseng (2010) denoted these two phenomena *perceptual blindness* and *attentional blindness*: perceptual blindness is a failure of perception caused by weak sensory signals that are difficult to distinguish from no signal at all, and attentional blindness is a failure of perception due to an inability to access sensory signals despite their presence. To probe whether the sense of confidence differs between these phenomena, Kanai et al. had observers perform six different detection tasks that manipulated stimulus visibility in different ways (contrast reduction, backward masking, flash suppression, dual task, attentional blink, and spatial uncertainty) and rate confidence in their responses. By analyzing confidence judgments, they demonstrated that target misses were *not* distinguishable from actual target absence for contrast reduction, backward masking, and flash suppression, but *were* distinguishable in the dual task, attentional blink, and spatial uncertainty paradigms. These results support the idea that metacognitive judgments may not index all detection failures equally, but much is still unknown about the relationship between metacognition and these visibility manipulations.

In the present study, we aimed to investigate perceptual metacognition in a detection task which manipulated stimulus visibility in two ways: (1) phase scrambling (PS), and (2) the attentional blink (AB). Phase scrambling is a manipulation whereby visual stimuli are scrambled to become unrecognizable, but the spatial frequency content of the image remains similar (Oppenheim, & Lim 1981; Thomson, 1999). The attentional blink is a phenomenon where a second target often goes unseen when it is presented between approximately 180 to 450 ms after the presentation of a first target in a rapid serial visual presentation (RSVP) (Raymond, Shapiro, & Arnell, 1992). Our rationale for using these conditions is built upon several neural and behavioral findings which indicate that PS and the AB likely result in different phenomenal experiences and different metacognitive assessments across the two conditions (Moratti et al., 2014; Dell'Acqua, Dux, Wyble, Doro, Sessa, Meconi, and Jolicœur, 2015; Kranczioch et al., 2005).

First, evidence indicates these manipulations rely on different neural systems. The capacity to consciously perceive a challenging PS picture appears to rest primarily upon processing in occipital circuitry (Moratti et al., 2014), whereas some “blinked” targets in AB paradigms cannot be seen because of disruptions within the frontal-parietal brain network (Dell’Acqua et al., 2015; Kranczioch et al., 2005). Second, behavioral results indicate that metacognitive differences emerge across task types (e.g., detection and discrimination) when employing visibility manipulations such as phase scrambling (Meuwese et al., 2014). In this study, metacognitive ability was assessed by plotting the receiver-operating characteristic (ROC) curves and computing the area under the curve (AUC) independently for each task-stimulus combination. It was shown that differences in metacognitive abilities emerged on “correct rejection” trials (i.e., trials where the subject correctly assesses that a target stimulus was *not* present) across different task conditions.

The results of Meuwese et al. (2014) may be explained by a violation of the assumption of equal variance in Signal Detection Theory (SDT). Specifically, in SDT it is assumed that on any given trial the subject’s nervous system represents the presence of either the nontarget (e.g., noise) stimulus or target stimulus as a random sample from a noisy representation of the information, and compares this sample to an internal criterion to make a perceptual judgment about stimulus presence or absence. The most common analysis in this framework assumes that the variances of the distributions representing the nontarget and target stimuli are equal (which is an assumption that is sometimes impossible to evaluate, as studies which do not record confidence reports do not have multiple points on the zROC function to assess variance asymmetry). When the variances are equal, standard measures of performance such as the area under the ROC curve (AUC) are unbiased. However, if the distributions representing the nontarget and target stimuli are *unequal*, as is often the case in detection tasks (Green & Swets, 1966; Macmillan & Creelman, 2005), then response-specific AUC measures are strongly influenced by interactions between the variance of the signal distribution and the placement of the response criterion, which can greatly influence results.

Critically, we hypothesized that this phenomenon in Signal Detection Theory (violating the assumption of the equal variance) may not only underlie the results of Meuwese et al. (2014), but could potentially explain differences in subjective experience between PS and AB visibility manipulations. Thus, we performed an experiment and simulations in which we aimed to assess the metacognitive ability of participants using a detection task with two conditions (phase scrambling and the attentional blink) to manipulate awareness of stimuli. These two conditions were partly chosen based on the aforementioned research by Kanai, Walsh, and Tseng (2010). Their research operationally defined metacognitive ability by computing a response-specific AUC for correct rejection and miss trials, and they showed that visibility manipulations cause differences in metacognitive ability for “No” responses (specifically, correct rejections: participants had higher levels of metacognitive ability for correct rejections in attentional blink trials compared to trials with physical degradation of stimuli), but similar metacognitive ability for “Yes” responses. These differences could be reflected by changes in specific mechanisms in SDT frameworks: the judgment that “there was nothing” for the AB seems phenomenally quite distinct from the judgment that “there was something I couldn’t recognize for sure” in PS, and identifying where this change emerges in SDT could provide novel insights about the relationship between SDT, metacognition, and phenomenology.

Therefore, in line with the results of Kanai et al. (2010), we hypothesized that there would be no differences in metacognitive ability (assessed by response-conditional Type 1 AUC values in our study) for the “Yes” response trials, but that there *would* be a difference for the “No” response trials. Specifically, we hypothesized that during AB trials, participants would be more aware of when they missed a stimulus and therefore be better at categorizing metacognitive judgements, which would not happen as often during PS trials. Finally, we hypothesized that this difference in metacognition would also be evident in a violation of the equal variance assumption of SDT in the experimental data. To anticipate, our hypotheses were supported by both our simulations and experimental results.

## Method

### Participants

In this study, 38 students at the University of California-Los Angeles (age range = 18–29 years, *M* = 20.29, *SD* = 1.99; 11 men) participated for partial fulfillment of course requirements. All participants had normal or corrected-to-normal vision, no history of neurological conditions, and gave written informed consent before participation. The experiment was approved by the UCLA Institutional Review Board. For the analyses, three participants (subject #3, #8, and #18) were excluded because they did not use the entire confidence rating scale as was instructed (e.g., if subjects use only one confidence rating for all trials, we cannot assess how confidence differs across our two conditions). In total, 35 students were included in the final analyses (age range: 18–29 years, *M* = 20.31, *SD* = 2.05; nine men).

### Stimuli

**Fig. 1:**
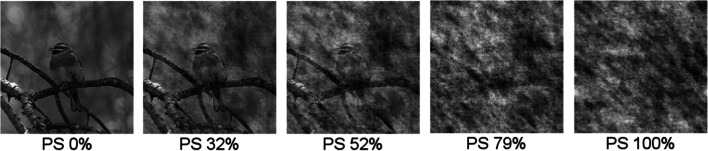
Examples of phase scrambling levels. This figure shows the spectrum of the amounts of phase scrambling used in the experimental task, from 0–100%. The rightmost image is the image that was used as the non-animal counterpart in all experimental tasks

The stimuli used in this experiment were from Meuwese et al. (2014), which included 600 grayscale animal photos from three categories: birds (200), cats (200) and fish (200). These stimulus categories were matched for low-level image statistics. For each image containing an animal, we created a non-animal-counterpart by fully randomizing the phase information in the image, effectively removing all object information (coherence) from the image (e.g., compare the leftmost and rightmost image in Fig. 1 to see the difference between an animal and its non-animal counterpart).

Phase-scrambling was also used to parametrically manipulate visibility in the PS task. The phase scrambled versions of these stimuli had similar low-level image statistics (e.g., overall contrast), but object information was gradually removed with increased levels of phase scrambling (see Fig. 1, left to right).

**Fig. 2:**
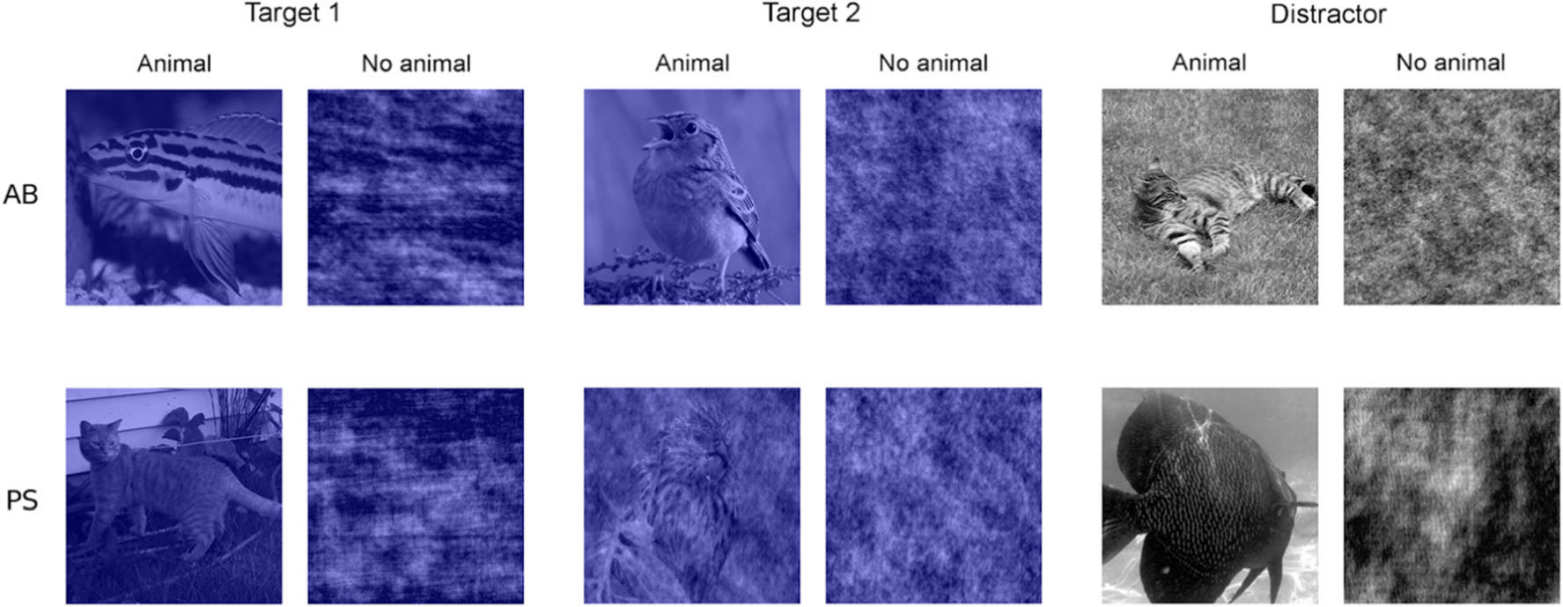
Examples of the targets and distractors for the attentional blink (AB) and phase scrambled (PS) stimuli. All blue stimuli are targets and all grayscale stimuli are distractors. AB stimuli are shown in the top row, and include examples of the first target (Target 1), examples of the second target (Target 2), and distractor stimuli. PS stimuli are shown in the second row, and include examples of the first target, examples of the second target (57.89% PS), and distractor stimuli

In both tasks, all distractor stimuli were gray and all target stimuli had a blue filter over the photo. Stimuli contained either animals or noise (i.e., a 100% phase scrambled photo) and were shown for 50 ms (see Fig. 2). The blanks in between stimuli lasted for 100 ms.

### Procedure

When participants came to the lab, they first filled out an eligibility checklist to confirm that they were 18 years or older, had normal or corrected-to-normal vision, did not have a neurological condition (e.g., epilepsy) and could fully comprehend the English language. After giving informed consent, they received specific instructions for the task. The task consisted of a rapid serial visual presentation (RSVP) paradigm and included two trial types: a short lag trial with 250 ms between target 1 and 2 (attentional blink, or “AB”), and a long lag trial with 850 ms between target 1 and 2 (phase scrambled, or “PS”). Thus, the design of this experiment has one independent variable (visibility) with two levels (AB vs. PS), but we note that the timing of the stimuli also differs across these two condition types by necessity.

**Fig. 3:**
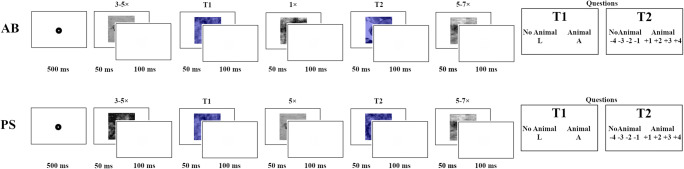
Representative trials for the attentional blink (AB) and phase scrambled (PS) trial types. In both conditions, following 500 ms of fixation, three to five distractors were shown, followed by the first target, which was either noise or a clear picture of an animal. After the first target, in the AB condition, one distractor was shown, immediately followed by the second target. In the PS condition, five distractors were shown before the second target was presented. The trial concluded with five to seven additional distractors being shown, followed by the question prompts asking whether the first target (T1) was an animal, and confidence regarding whether the second target (T2) contained an animal. All stimuli were shown for 50 ms and all blank screens for 100 ms. The response screens were shown until a key was pressed

Each trial started with a fixation dot for 500 ms, after which the RSVP started (Fig. 3). First, three to five gray distractors were presented (the number of three to five was pseudo-randomly selected every trial). Then, the first target (T1) was shown in blue. In both conditions, T1 was either fully intact if it contained an animal (non-scrambled) or 100% phase-scrambled if it did not contain an animal (i.e., if it was noise). Therefore, T1 did not differ between trial types. Next, a variable number of gray distractors were shown. After these distractors, the second blue target (T2) appeared. For AB trials there was only one gray distractor between T1 and T2, while for PS trials there were five gray distractors between T1 and T2. For AB trials, T2 was either a blue non-PS picture (animal) or a blue, 100% phase-scrambled picture (no animal). For PS trials, the second target was either a blue partially PS picture (animal), or blue 100% scrambled picture (no animal). After the second target, five to seven gray distractors (pseudo-randomly assigned using the np.random.choice function from the SciPy library (Jones, Oliphant, & Peterson, 2001)) appeared. Finally, the questions for the targets appeared.

Participants initially had to decide whether the first target (T1) was an animal or not and press the corresponding key. Then participants had to indicate whether the second target (T2) contained an animal or not, while simultaneously indicating their confidence about that decision with four levels of certainty (8-point scale: 8 entirely certain not an animal, 7 most likely not an animal, 6 probably not an animal, 5 unsure not an animal, 4 unsure an animal, 3 probably an animal, 2 most likely an animal, 1 entirely certain an animal). This response method combines the classical “Type 1” judgment (assessing whether or not the target contained an animal) with a “Type 2” judgment (evaluating the confidence one has in the judgment of whether the target contained an animal). Participants were explicitly instructed that the first response should only reflect their decision about the first target (T1) and that the second response should only reflect their decision about the second target (T2) that appeared in each sequence.

Participants began the experimental task by completing practice blocks designed to yield equal performance for the AB and PS trial types. The first block consisted of 54 trials, including 36 AB trials and 18 PS trials, to identify an average percentage correct for the AB trials (PS trials were included so participants would not learn to expect the exact time the stimulus would appear, but were excluded from further analysis). If the performance on the AB trials was not high enough (i.e., was lower than 65% correct), the participant had to redo this block until their performance was higher than 65%. The second block (108 trials; 72 PS trials and 36 AB trials) used a staircasing procedure (QUEST) (Watson & Pelli, 1983) to find a degree of PS such that the percentage correct between the AB and PS trial types was approximately equivalent.

During the main experiment, participants completed eight blocks of 72 trials, for a total of 576 trials. Within each block, AB and PS trials were randomly interleaved, and there was an equal number of each trial type. To ensure performance was matched in all blocks for T2 responses for the AB and PS trials, whenever the percentage correct on PS trials became more than five percentage points higher than the percentage correct on AB trials, the PS level was raised by 1/19 (yielding 20 possible PS levels as 0% was also included). If the percentage correct on long lag trials got worse than ten percentage points below the short lag, the PS level was lowered by 1/19.

## Results

To evaluate whether the objective (type 1) difficulty between trial types was successfully matched, we performed a paired-samples t-test in the following manner: first, we selected the subset of trials which included a correct T1 response. (This is because in AB trials, it is easier to have the T2 response correct if T1 was unattended or ignored; thus, we aimed for consistency across trial types.) Then, we selected the type 1 (animal/no animal) judgments from T2 responses and performed a paired-samples t-test on the percentage correct between AB and PS trials. Results showed that performance for T2 (when T1 was correct) was similar for the two trial types (AB trials: 70.00% correct [range = 54.39–81.07, *SD* = 6.1], PS trials: 69.28% correct [range = 57.75–79.21, *SD* = 5.3], *t*(34) = 1.40, *p* = .17). For full transparency, we note that participants had on average 2% more T1 correct on PS trials compared to AB trials (PS: 91.84% correct, *SD* = 8.0%; AB: 89.69% correct, *SD* = 9.3%; *t*(34) = -3.67, *p* < .001).

**Fig. 4:**
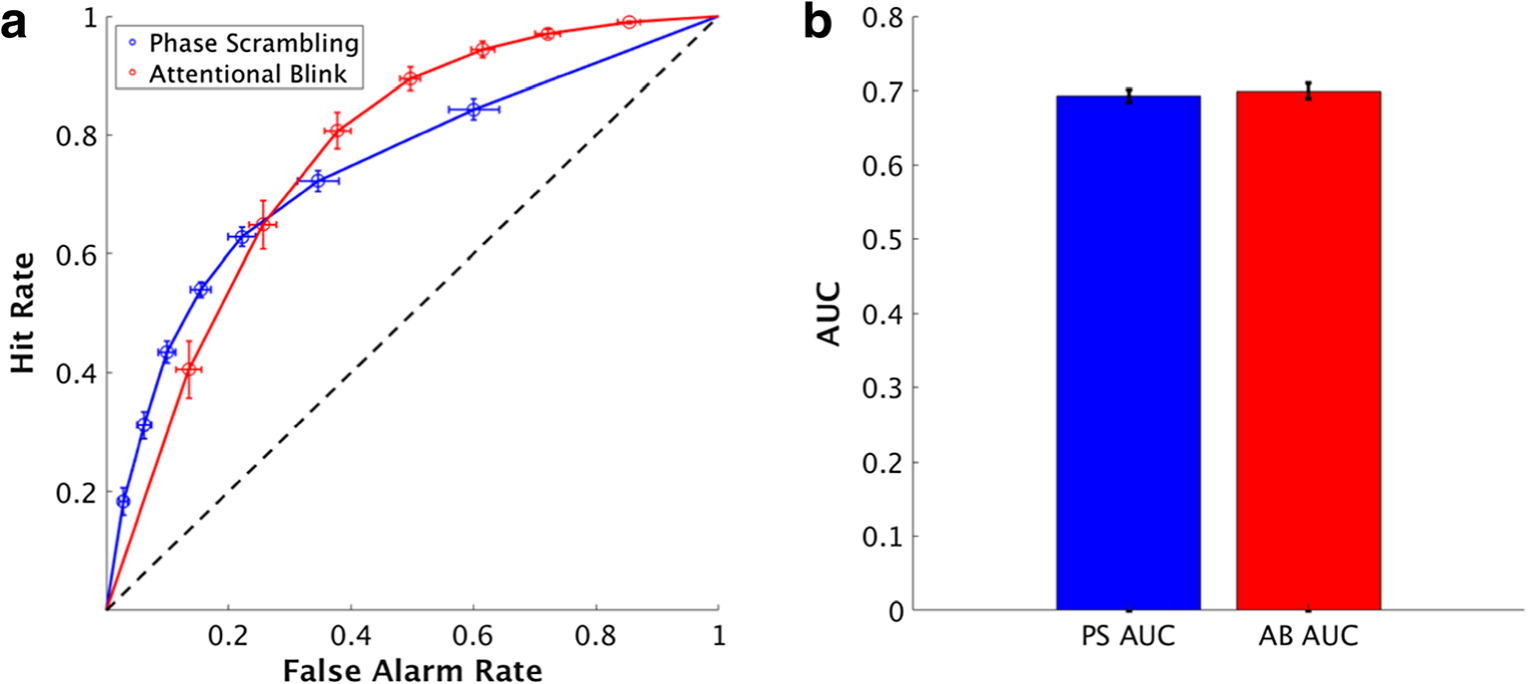
Pseudo Type-1 receiver-operating characteristic (ROC) curves and area under the curve (AUC) values for the attentional blink (AB) and phase scrambled (PS) trials. (**a**) The ROC curves for each condition. Each curve plots the average data across all subjects. Each dot on the graph represents data from a different confidence rating; specifically, each dot represents a different decision criterion (for a given confidence rating) and the corresponding percentage of hits and false alarms. The black dotted line provides a reference for chance-level performance. Error bars represent SEM across subjects. (**b**) The average AUC values for the PS and AUC conditions. These AUC bars plot the averaged AUC values for individual subjects’ curves, with error bars denoting SEM across subjects

To visualize the behavioral responses, we first plotted pseudo Type 1-ROC curves, which were formed by using each confidence rating as a decision criterion for performing the detection task and plotting the corresponding false alarm and hit percentages for each of these points (Fig. 4a). A Wilcoxon signed-rank test of the AUC values (computed using MATLAB’s “perfcurve” function) between trial types revealed that the values were not significantly different from one another (z = -1.16, *p* = .24, Fig. 4b), although the shapes of the curves were quite distinct. Overall, this provided further support for matched type 1 performance between the PS and AB trial types.

**Fig. 5:**
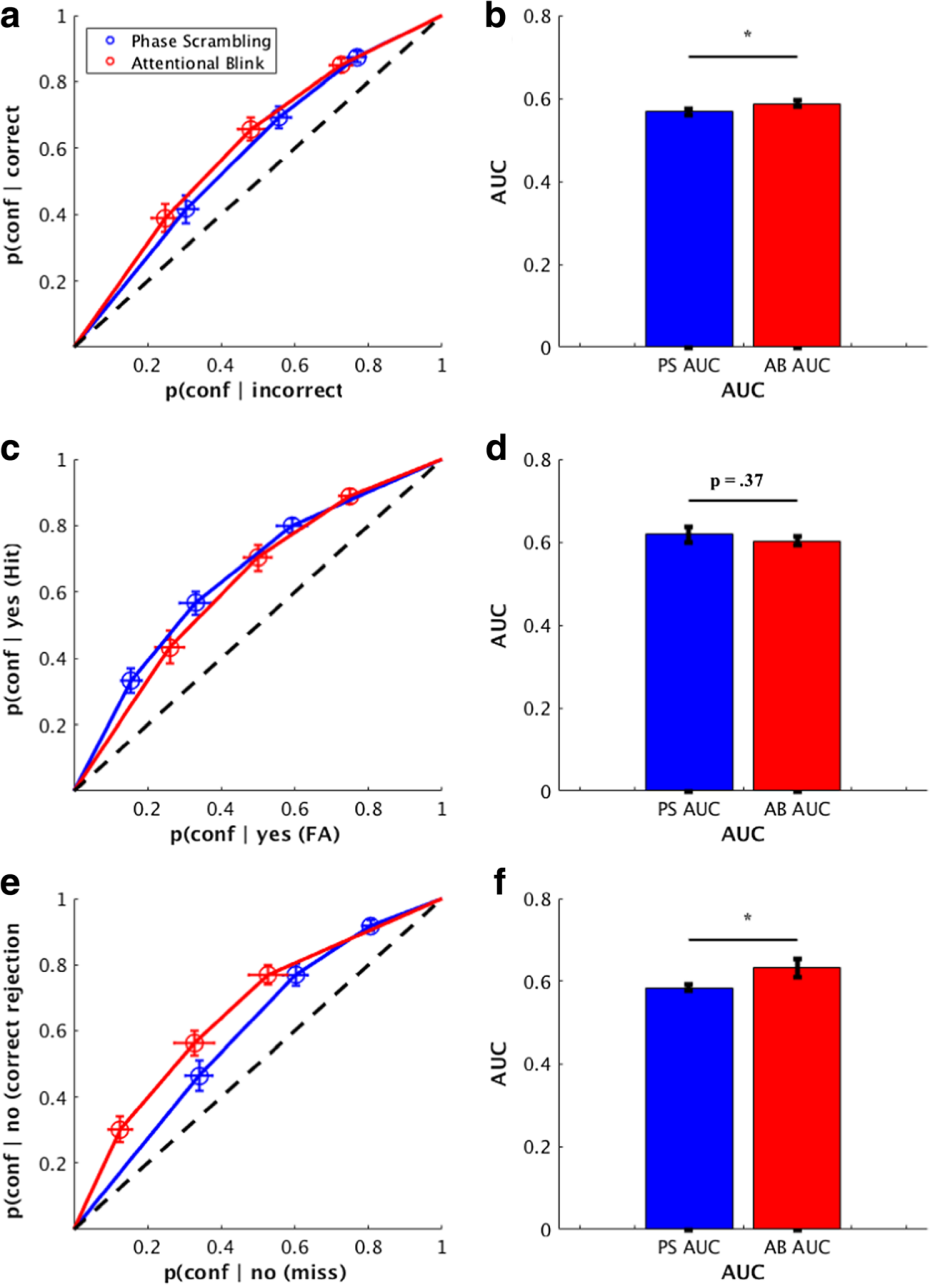
Response-conditional receiver-operating characteristic (ROC) curves using across-subject averages. (**a-b**) The probability for each confidence judgment given either a correct or incorrect response. Correct responses were composed of the summed counts for correct rejections and hits for each confidence level, and incorrect responses were composed of the summed counts for misses and false alarms across each confidence level. The ROC curve plots the average results across all subjects, and the AUC bars plot the averaged AUC values for each individual subjects’ curves. Error bars represent the standard error of the mean across subjects. (**c-d**) The probability of each confidence judgment given a Yes response. The y-axis shows this probability for Yes responses that were correct (hits), and the x-axis shows this probability for the Yes responses that were incorrect (false alarms, FA). Conventions are the same as panels A-B. (**e-f**) The probability of each confidence judgment given a No response. The y-axis shows this probability for no responses that were correct (correct rejections), and the x-axis shows this probability for no responses that were incorrect (misses). Conventions are the same as panels A-B. *p< .05

Next, we analyzed whether there was a general difference for metacognitive ability between trial types using the same methods as (Meuwese et al., 2014); namely, ROC curves from conditional responses (correct/incorrect trials, yes responses, and no responses). Figure 5a shows the ability of confidence ratings to distinguish between correct and incorrect responses, using the data from all subjects. There was a significant difference for average confidence ratings to better distinguish correct and incorrect trials in AB trials than in PS trials, as shown by a Wilcoxon signed-rank test of the area under the curve (AUC) values (z = -2.09, *p* =.036). To explore the difference between trial types in more detail, type 2 ROC curves were made for the “Yes, animal present” responses (false alarm and hit trials). These curves (Fig. 5c and d) showed that performance on these trials was quite similar between the PS and AB conditions (z = 1.15, *p* = .25). However, the type 2 ROC curves of the “No, animal not present” responses (misses and correct rejections (CR)) (see Fig. 5e and f) did indicate a difference in AUC values between the AB and PS trial types (z = -2.00, *p* = .046). Thus, subjects were better at using metacognitive judgments in AB trials to distinguish between correct and incorrect trials for “No” responses compared to when they were using metacognitive judgments in PS trials.

Interestingly, the higher metacognitive performance in AB trials compared to PS trials was slightly modulated by whether or not an animal was present in T1. For the AB Trials, an analysis of all responses to T2 (both yes and no), indicated there was a difference that was almost significant between T1 present and T1 absent trials (*t*(34) = 1.97, *p* = .06). Breaking this down further, we analyzed differences in metacognitive ability for the T2 yes and T2 no responses separately. Participants had better metacognitive ability for T2 yes responses when T1 *did* contain an animal (*t*(34) = -2.18, *p* = .04). Conversely, participants had slightly worse metacognitive ability for T2 no responses when T1 did *not* contain an animal (*t*(34) = 2.21, *p* = .03). This indicates that metacognition for the AB may differ slightly based on whether first image is a target or not. Similar findings did not emerge for the PS trials: measured through type 2 AUC values, participants’ metacognitive ability did *not* differ between T1 animal present and absent trials, when all responses on T2 were taken into account (*t*(34) = -0.07, *p* = .94), when only yes answers (“there is an animal in T2”) were analyzed (*t*(34) = 1.08, *p* =.29) and also when only no answers were analyzed (“there is *no* animal in T2”) (*t*(34) = 0.57, *p* = .57).

**Fig. 6:**
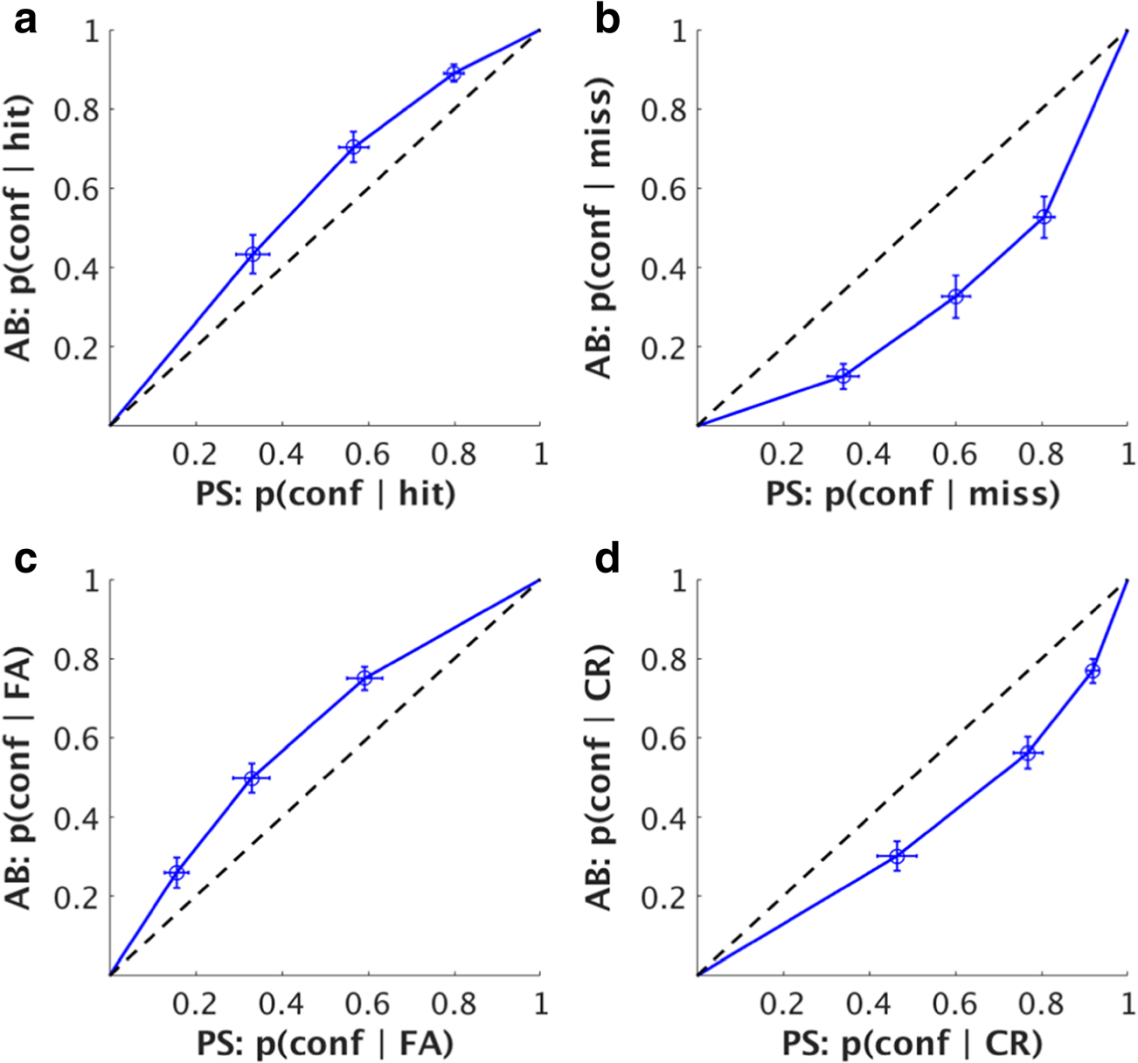
Receiver-operating characteristic (ROC) curves for the PS and AB conditions for all Type-1 responses using across-subject averages. Each point on the graph represents the criterion for a given confidence rating. (**a**) Hits, (**b**) Misses, (**c**) False Alarms (FA), (**d**) Correct Rejections (CR)

We then sorted our data to determine if differences in confidence between the AB and PS conditions were driven by a specific Type 1 stimulus-response combination. Figure 6 displays ROC curves for each Type 1 response, including (A) hits, (B) misses, (C) false alarms, and (D) correct rejections. As can be seen in the results, a clear pattern emerges: “yes, target present” responses (left column) lead to a stronger confidence profile in AB compared to PS, whereas “no, target absent” responses (right column) lead to under-confidence in the AB compared to PS, regardless of whether they were correct in their judgement of target presence or absence.

Interestingly, our results conceptually replicate several findings by Meuwese et al. (2014). In their study, they reported significant differences between the target detection tasks participants performed (i.e., categorization and detection). They showed that metacognitive ability was higher for categorization compared to detection (based on AUC values plotting correct vs. incorrect responses) across all trials, and that this difference was primarily driven by differences in “No” response trials (their Fig. 2). Additionally, they did not show a significant difference in metacognitive ability between tasks based solely on the “Yes” response trials. The authors concluded that metacognitive ability is better in tasks that rely on positive evidence (categorization) than in tasks that rely more strongly on absence of evidence (detection).

**Fig. 7:**
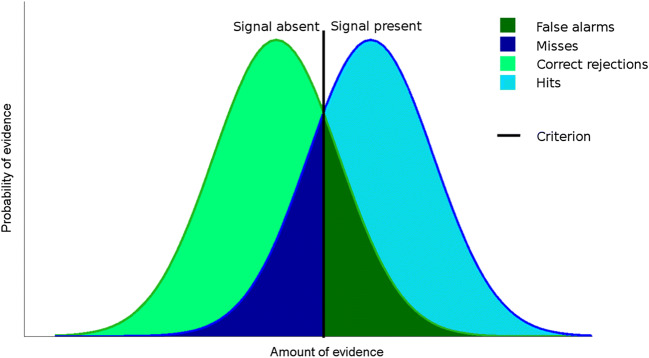
Signal Detection Theoretical (SDT) framework. In SDT, it is assumed that on each trial of a detection experiment, an observer’s internal representation can be modeled as a sample drawn from either a “stimulus-present” (blue) or “stimulus-absent” (green) distribution. To make a decision about stimulus presence or absence, this sample is then compared to an internal criterion; if the sample is above the criterion, the participant responds “signal present,” and if the sample is below the criterion, the participant responds “signal absent.” This results in four different trial types: trials where the stimulus was present and the subject correctly responds that it was presented (hits, light blue); trials where the stimulus was present and the subject says that it was not (misses, dark blue); trials where the stimulus was not present and the subject says that it was not (correct rejections, light green), and trials where the stimulus was not present, but the subject says that it was (false alarms, dark green)

However, the quantitative framework used by Meuwese and colleagues to analyze their results, Signal Detection Theory (SDT), provides a possible alternative explanation of why differences in metacognitive ability may have emerged across tasks. SDT is typically used to analyze perceptual decisions that are made in uncertain or ambiguous situations (Wickens, 2002). In this framework (Fig. 7), whenever a stimulus is presented, it is assumed that an internal response is generated which encodes the stimulus (e.g., either signal or noise), and this response represents a sample from a distribution with some amount of variability. On each trial, the observer compares the strength of the evidence they obtained to an internal criterion, and makes a judgment as to whether this stimulation originated from a signal present in the environment or random noise. For a detection task (e.g., “is ‘A’ present or not?”) the decision-making process can be represented by two curves, one capturing ‘A’ absence (e.g., a noise trial) and one capturing ‘A’ presence (e.g., a signal trial). This yields four different possibilities for type 1 performance: hits, misses, false alarms, and correct rejections.

**Fig. 8:**
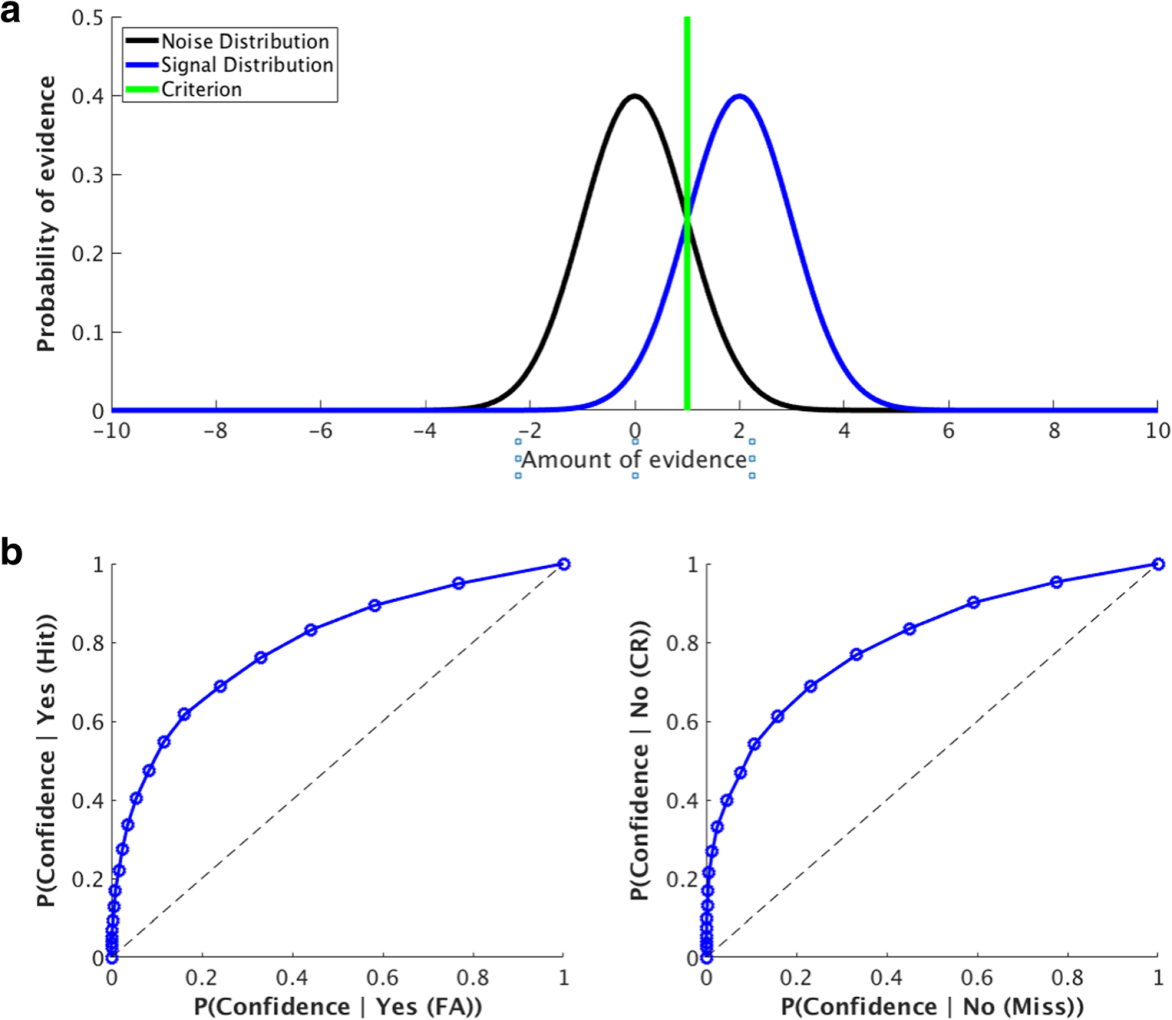
Simulation of a detection task with equal variances for the signal and noise distribution, using standard signal detection theoretic tools. (**a**) Graphical representation for stimulus encoding and perceptual decisions in this framework. Shown here are two distributions, representing the encoding of a sensory signal (blue) and the encoding of noise (black). On any given trial, either a stimulus or noise is presented, and the observer compares a sample drawn from one of these two distributions to the internal criterion (green) to make their perceptual judgment about stimulus presence/absence. (**b**) Receiver-operating characteristic (ROC) curves for trials where subjects responded “yes, stimulus present” (left) or “no, stimulus absent” (right). The different confidence ratings in this simulated experiment (20 total) are shown in blue. The closer the point on the blue curve is to the origin, the higher the given confidence rating (blue dots). Whenever the curve is above the black diagonal, it indicates that the metacognitive responses of the observer can distinguish correct from incorrect trials

In many studies employing SDT, it is assumed that the signal and noise distributions have equal variances. To simulate the results from a detection task where the signal and noise have equal variance, we can sample 10,000 trials from the noise distribution (mean 0, var 1) and 10,000 trials from the signal distribution (mean fixed at 2 for this simulation, var 1), and then sweep the criterion along the x-axis (from -2 to 4, using 20 different criteria for confidence) to plot full ROC curves for hits versus false alarms, and correct rejections versus misses. The results from this simulation are shown in Fig. 8. In the “Yes” response ROC plot (Fig. 8b, left), as in Meuwese et al. (2014), the leftmost inflection point is the proportion of Yes/Correct (i.e., hits) responses for which the highest confidence rating was given versus the percentage of Yes/Incorrect (i.e., false alarms) responses with the highest confidence rating. The “No” response ROC plot (Fig. 8b, right) follows the same convention. With equal variances for these two distributions, these type 1 response-conditional ROC curves have a characteristic shape: that is, ROC curves which plot the probability of confidence given a hit versus the probability of confidence given a false alarm, and ROC curves that plot the probability of confidence given a correct rejection versus the probability of confidence given a miss, have similar shapes.

**Fig. 9:**
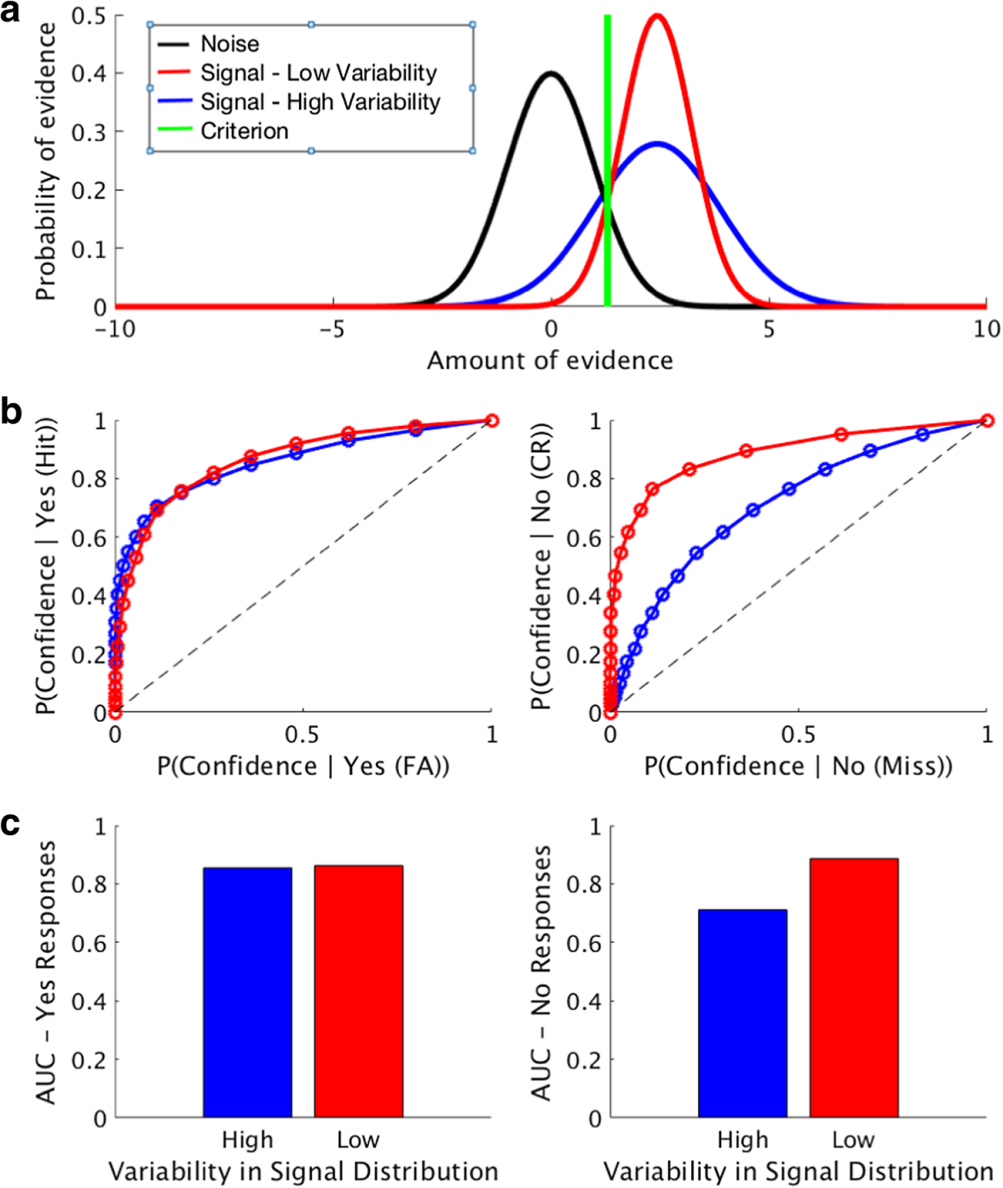
Theoretical simulation of a detection task with unequal variances for the signal and noise distributions. (**a**) Graphic representation of the type 1 decision space. Shown here is an example where the variability of encoding of the sensory signal is either lower (blue) or higher (red) than the precision of encoding the noise signal (black). The distributions differ in standard deviation (.8 & 1.43 for red and blue, respectively), but have the same mean (2.44). Perceptual decisions are made by comparing the sample drawn to an internal criterion (shown in green). (**b**) Response-conditional ROC Curves for simulated data for trials where the subject responded “yes, stimulus present” (hits and false alarms) or “no, stimulus absent” (misses and correct rejections). As can be seen in this example, the ROC curves are quite similar for both the low-variance and high-variance cases for trials where the subject responses “yes, stimulus present,” indicating variance of the signal distribution has minimal impact on this measure. However, the variance of the signal distribution has a much greater influence on the trials where the subject responded “no, stimulus absent,” illustrating the importance of testing the assumption of equal variances when computing AUC values for this measure. (**c**) Area under the curve for the “yes” response trials (hits and false alarms) and area under the curve for the “no” response trials (correct rejections and misses)

However, the assumption of equal variance between the signal and noise distributions can be violated. In circumstances where the variances of these two distributions differ, distinct patterns emerge for the response-conditional ROC curves for stimulus absence, which can directly influence AUC magnitude (Fig. 9). Critically, violation of this assumption strongly influences the ROC curves for “signal-absent” trials, illustrating the importance of evaluating this assumption before applying SDT analyses to data.

**Fig. 10:**
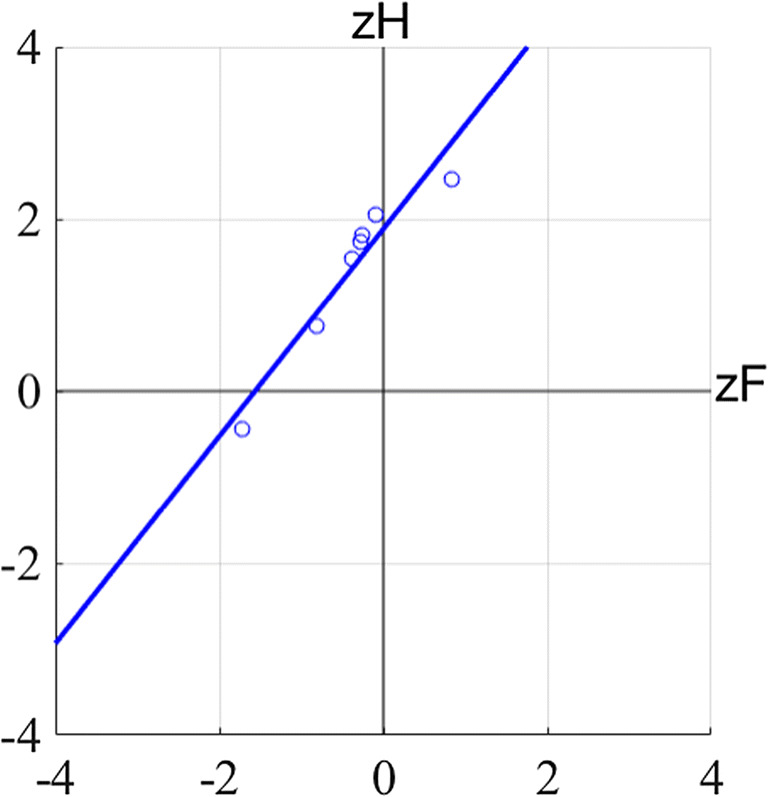
zROC space for one representative subject. Shown in this figure are the z-scored false alarm rates and hit rates for the attentional blink (AB) condition for a representative subject, with the linear model fit to these points

To explicitly evaluate whether the equal variance assumption was violated for the AB and the PS trials in our experiment, Type 1 zROC analyses were performed (Fig. 10). Specifically, the hit and false alarm rates of the type 1 ROC analysis were z-scored for each participant. A linear line was then fit to each participant’s data through the points in zROC space using MATLAB’s “polyfit” function, yielding an estimated slope and intercept for each subject.

If the slopes of these lines in zROC space are near 1, this supports the idea that the equal variance assumption has been met; if the slopes of these lines are different from 1, it is likely that the equal variance assumption has been violated. However, as noted by (Wickens, 2002), “any theoretical model, once its parameters are estimated, implies a comparable table of predicted frequencies. If the observed frequencies are similar to the predicted frequencies, then the model fits, and if they differ, the model fails (p.213).” Therefore, we also performed chi-square goodness of fit tests, comparing our observed frequencies to the expected distributions under the equal-variance model and unequal variance model, and conducted a hierarchical model comparison to see if one model was significantly better than the other.

**Fig. 11:**
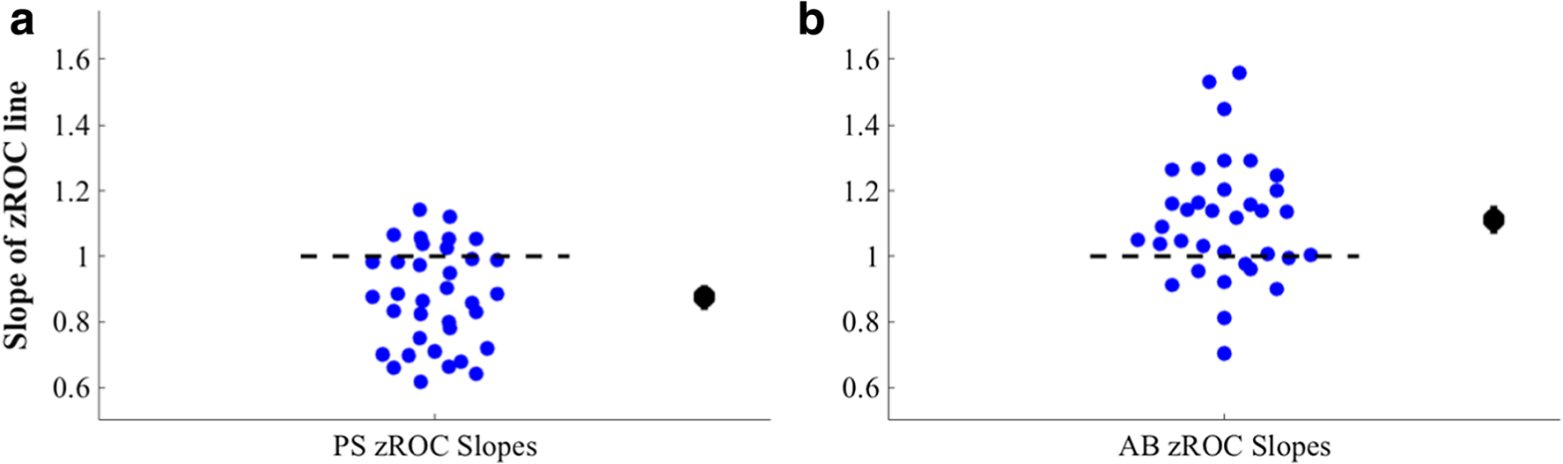
Slopes of zROC lines. (**a**) Phase scrambled (PS) trials. Shown in this panel are the slopes of the zROC lines for each individual subject. In general, lines that deviate from a slope of 1 (the black dotted line) indicate that that the equal variance assumption has been violated. The black dot on the right-hand side indicates the average slope of the zROC lines, with SEM across subjects. (**b**) Attentional blink (AB) trials, with the same conventions as panel A

Results confirmed the hypothesis that for many subjects both AB and PS trial types were marked by unequal variances for the signal and noise distributions (see Fig. 11). For PS trials, the slopes on average were *below* 1 (mean = 0.87, p < .0001), and 17 of the 35 subjects were better explained by a model that assumed unequal variances (chi-square value > 3.84 for these subjects). For AB trials, the average slope of the zROC lines was *above* 1 (mean = 1.11, p < .01), and 12 of the 35 subjects were better explained by a model assuming unequal variances (chi-square value > 3.84 for these subjects).

**Fig. 12:**
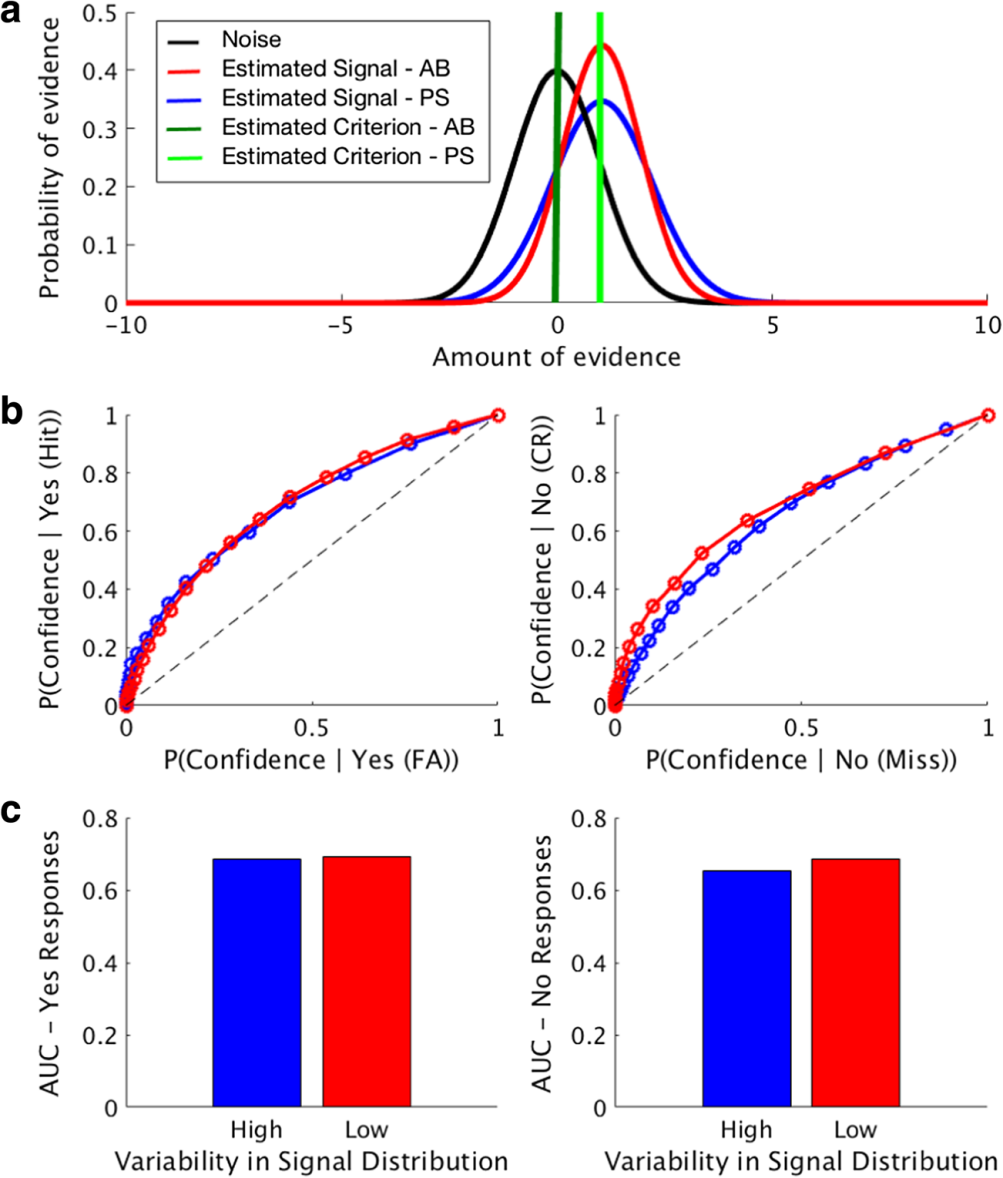
Detection task results based on experimental data, using the estimated slopes, intercepts, and criteria, averaged across all participants. (**a**) Graphic representation of the decision space. Shown here are the estimated variances for the signal distributions for the phase scrambling (PS; blue) and attentional blink (AB; red) conditions using the average values from the slopes and intercepts of the zROC lines for each participant. We also include the average criteria for the AB (dark green) and PS (light green) conditions. (**b**) Receiver-operating characteristic (ROC) curves using the average zROC slope, zROC intercept, and criterion across subjects for the PS (blue) and AB (red) “yes, stimulus present” (hits and false alarms) or “no, stimulus absent” (misses and correct rejections). As can be seen in this example, the ROC curves are quite similar for both the low-variance and high-variance cases for trials where the subject responses “yes, stimulus present,” indicating variance of the signal distribution has minimal impact on this measure. However, the variance of the signal distribution has a much greater influence on the trials where the subject responded “no, stimulus absent,” illustrating the importance of testing the assumption of equal variances when computing AUC values for this measure. (**c**) Area under the curve for the “yes” response trials (hits and false alarms) and area under the curve for the “no” response trials (correct rejections and misses) for the PS (blue) and AB (red) conditions

In a final analysis, using the estimated slopes and intercepts for the AB and PS trials, we computed the average slope and average intercept across all subjects to determine if our data supported the hypothesis of unequal variances for the signal and noise distributions for the two conditions. The amount of simulated confidence criteria was set to 20 and were evenly placed around the criterion (C) of the participant that was simulated, half on the right of C and half on the left. As can be seen in Fig. 12, the estimated variance for the distributions of both the AB and PS conditions appear to violate the assumption of equal variance, and using these values (which includes estimates for the average mean for these distributions across subjects), this scenario can produce matched ROC curves and AUC values for “yes” responses (Fig. 12b, left, Fig. 12c, left) and mismatched ROC curves and AUC responses for “no” responses (Fig. 12b, right, Fig. 12c, right). Though the overall magnitude of the effect in our data is smaller than the demonstration in Fig. 10, this is to be expected: as shown in Fig. 11, while some subjects violate the assumption of equal variance with slopes that are different from 1, other subjects’ results are modeled quite effectively with distributions with equal variance, as slopes are near 1. Overall, this analysis supports the hypothesis that unequal variances can drive the observed effects, and is further verified by its congruence with results from Fig. 5.

## Discussion

Previously, it has been shown that metacognitive ability is higher for responses in a categorization task compared to a detection task, and that this difference is primarily driven by performance on trials in which subjects reported that a target stimulus was not presented on a given trial (Meuwese et al., 2014). We hypothesized that this previous finding may be accounted for by violations of the assumption of equal variance in signal detection theory, and performed simulations and an experiment to replicate these results and demonstrate possible theoretical underpinnings of this reported effect. Through our modeling simulations, we find that, indeed, when the variance of “signal present” distribution is different from the “signal absent” distribution, this has a minimal impact on the area under the ROC curve for trials were participants report that a target stimulus was presented, but a significant impact on the ROC curve for trials when participants report that a stimulus was *not* presented (Fig. 9). In our experiment, we had subjects perform a detection task using two conditions shown to produce matched type 1 performance and different levels of metacognition on trials with reported stimulus absence (Figs. 4 and 5) and find that these conditions violate the assumption of equal variance in signal detection theory, and therefore can at least partially explain previous results (Figs. 10, 11 and 12).

Kanai et al. (2010) offered one possible explanation of what may be driving these effects: differences in metacognitive judgments may be due to an “awareness availability.” The awareness in AB or short lag trials might be in an all or none fashion (as suggested by Sergent & Dehaene, 2004), while the PS or long lag trials may be more gradual. In other words, whereas attentional blindness is subjectively an all-or-none phenomenon (an observer either notices or doesn’t notice the second target, i.e. it is either present or absent in the awareness), the PS manipulation causes difficulties in recognition/categorization of a supraliminal (and thus clearly perceptible) target. We find some evidence to support these claims: as shown in Fig. 6, “yes” responses (left column) lead to a stronger confidence profile during attentional blindness compared to phase scrambling (regardless of whether the yes response is correct), whereas “no” responses (right column) lead to under-confidence during attentional blindness compared to phase scrambling. As “signal” trials are degraded in phase scrambling, it seems reasonable that subjects have more confidence in hits during attentional blindness than during phase scrambling, and interestingly, this effect carries over to false alarms. Conversely, the performance drop for “no” responses during attentional blindness is caused by an inability to access the target, and this makes participants less confident that their response is correct when they miss a target or report absence of a trial, compared to phase scrambling. Thus, at some level subjects seem to “know” when they miss a trial due to attentional blindness when compared to phase scrambling, as a miss is caused at a higher level in the decision-making hierarchy.

While our results provide evidence for our conclusion, there were some limitations in this study. For example, as noted by Maniscalco and Lau (2014), constructing pseudo-type 1 ROC curves based on confidence judgments risks confounding type 1 and type 2 sensitivity. If separate mechanisms influence type 1 and type 2 judgments, it is possible that asymmetries emerge at the metacognitive level (due to response-conditional changes in type 2 sensitivity), which is then mimicked by a type 1 unequal variance model. As shown in the simulations in Maniscalco and Lau (2014), pseudo-type 1 ROC curves with slopes that are different from one can be generated by an equal variance model with differences in response-specific metacognition. Thus, while our results suggest this variance asymmetry, it is potentially also consistent with an equal-variance model but with response-conditional metacognitive noise.

Moving forward, additional findings to strengthen the unequal variance explanation of yes/no asymmetry in metacognition could come in using an independent method to shift the type 1 criterion (i.e., other than confidence ratings), such as payoff asymmetries, changes in stimulus base rates, or instructions in separate sessions. From these data, one could fit the variance ratio to the type 1 ROCs from this session, and ask whether it predicts the yes/no type 2 ROC asymmetry in the confidence rating session. If it does, then this is strong evidence that the metacognitive asymmetry emerges from unequal variance at the type 1 level that is manifest whenever there is a shift in type 1 criterion.

Another important limitation in the study was our inability to staircase the AB trials to a fixed percentage. Staircasing would involve changing the stimulus duration in general or contrast of the AB (second) target. Both would be theoretically difficult. Changing stimulus duration would also have to be paralleled in the PS trials, but then it would be difficult to staircase the long lag trials since the staircased variable (level PS) difficulty would depend on the (slightly) changing stimulus duration. Changing the contrast of the second target in AB trials would also be theoretically difficult, since then the AB trials would include both perceptual and attentional blindness manipulations (Kanai et al., 2010), and the rationale was to include one perceptual and one attentional blindness manipulation. Nevertheless, the analyses showed that the average percentages were around 70% correct across both conditions, which is near threshold. We also think that requiring subjects to make the same type of confidence judgment for both T1 and T2 trials would be worthwhile, as the current procedure might have induced the heightened metacognitive sensitivity to their T2 performance, which could in turn bias the results.

A final limitation to note is the lack of a fitting control trial type. We considered including trials in which the second target would be outside the AB timespan (as with the long lag) and without phase scrambling. However, such trials would be substantially less challenging or difficult and could have influenced how the confidence ratings would be distributed across trials of the same difficulty. The control trials would be more clearly visible, and thus also would have received higher confidence ratings while the experimental trials (AB and PS) would have gotten the lower confidence ratings. For this reason, we did not include separate control trials.

Nonetheless, we consider these results informative. As we demonstrate in our simulations and experimental data, these findings stress the importance of assessing whether the equal variance assumption has been violated when analyzing detection-task data. A suggestion for future research is to combine this paradigm with EEG, to see if there is a difference in how well a decoder can categorize animal targets from no-animal targets for both trial types (short lag and long lag) and if this correlates with the metacognitive judgments. Finally, we also suggest that researchers compare conditions using manipulations other than attentional blink and phase scrambling, to see if these same trends would emerge.
